# Supplementary material for: Synthesis and evaluation of protein-based biopolymer in production of silver nanoparticles as bioactive compound versus carbohydrates-based biopolymers
Source: R Soc Open Sci. 2020 Oct 21;7(10):200928. doi: 10.1098/rsos.200928 (PMC7657912; doi:10.1098/rsos.200928)
Supplement: Charts of TGA and FTIR [file rsos200928supp1.zip › TGA-IR charts/FTIR sodifum caseinate-AgNPst.pdf]

Peak Find – caseinate-AgNPs.jws

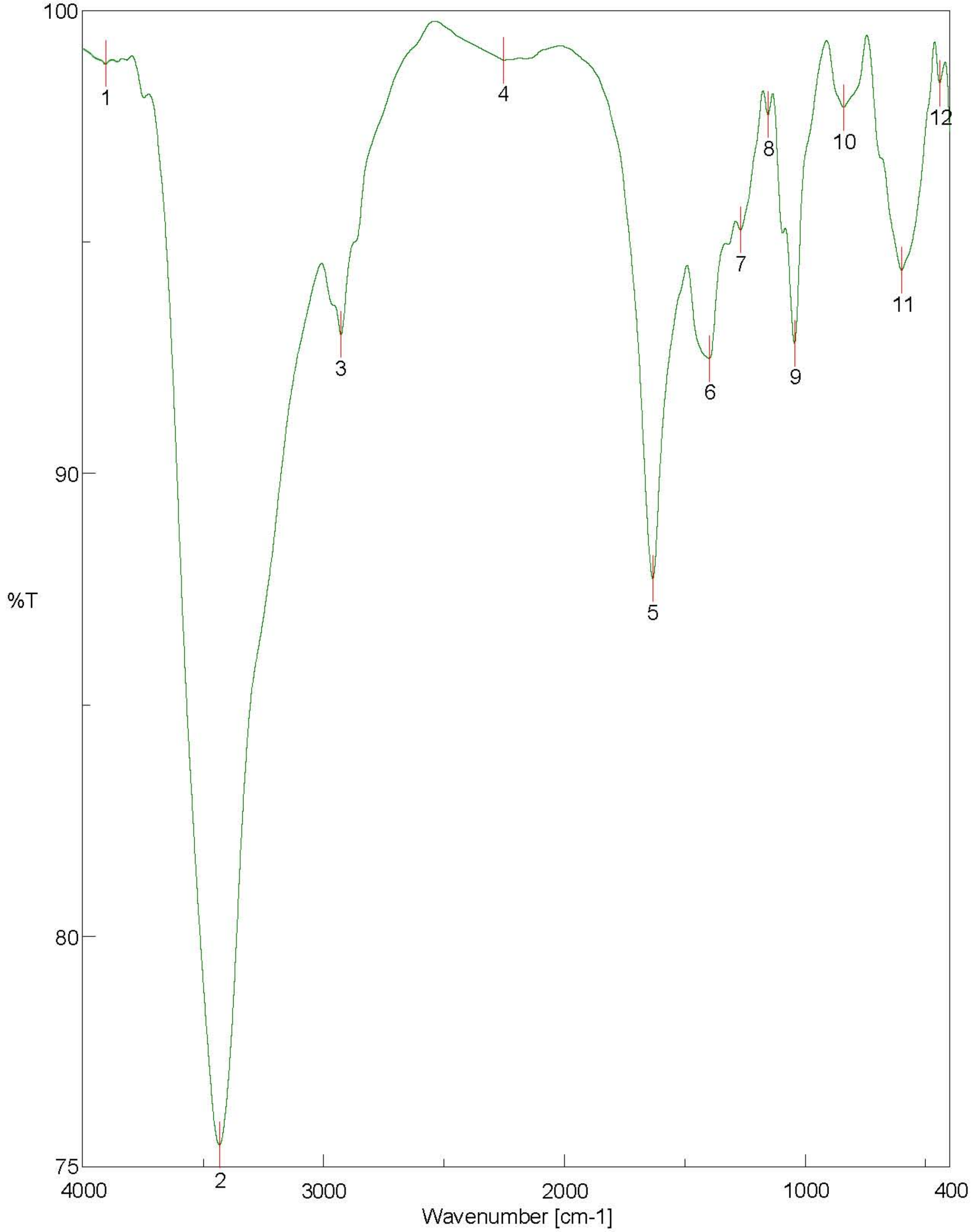

[Result of Peak Picking]

No.	Position	Intensity	No.	Position	Intensity	No.	Position	Intensity
1	3903.22	98.8574	2	3432.67	75.4798	3	2927.41	92.9966
4	2253.41	98.9302	5	1633.41	87.7276	6	1399.1	92.4803
7	1268.93	95.2576	8	1155.15	97.7548	9	1045.23	92.8138
10	840.812	97.9122	11	599.753	94.39	12	442.583	98.4402
