# Supplementary material for: Synthesis and evaluation of protein-based biopolymer in production of silver nanoparticles as bioactive compound versus carbohydrates-based biopolymers
Source: R Soc Open Sci. 2020 Oct 21;7(10):200928. doi: 10.1098/rsos.200928 (PMC7657912; doi:10.1098/rsos.200928)
Supplement: Charts of TGA and FTIR [file rsos200928supp1.zip › TGA-IR charts/TFA Sodium caseinatet.pdf]

Sample: caseinate
Size: 14.4020 mg
Method: Ramp

DSC•TGA

File: C:...\SDT\feb2015\Altaf\7•4•2019\Ca.001
Operator: ahmed t
Run Date: 02•Apr•2019 03:26
Instrument: SDT Q600 V20.9 Build 20

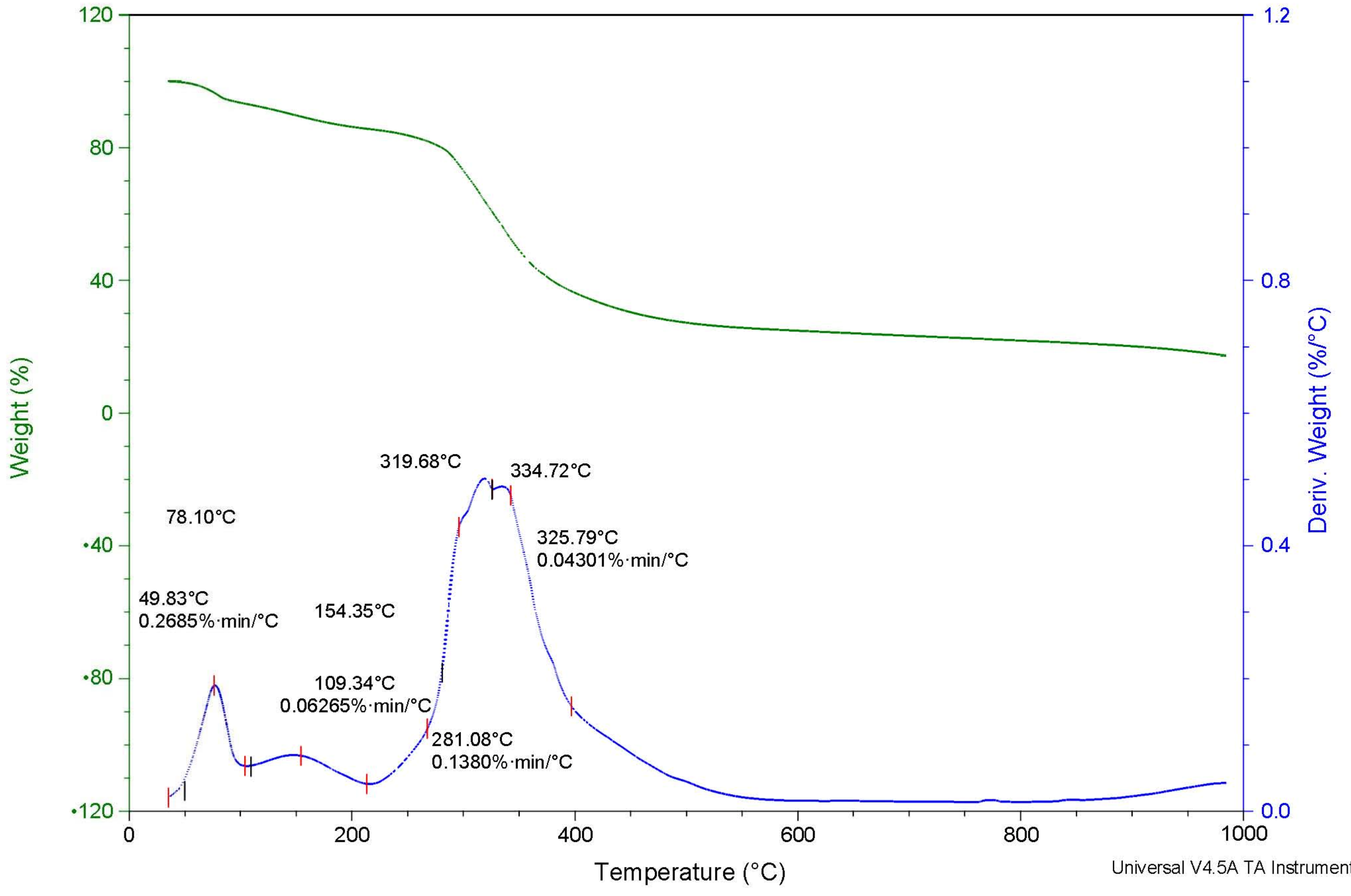

Universal V4.5A TA Instruments
