# Supplementary material for: Synthesis and evaluation of protein-based biopolymer in production of silver nanoparticles as bioactive compound versus carbohydrates-based biopolymers
Source: R Soc Open Sci. 2020 Oct 21;7(10):200928. doi: 10.1098/rsos.200928 (PMC7657912; doi:10.1098/rsos.200928)
Supplement: Charts of TGA and FTIR [file rsos200928supp1.zip › TGA-IR charts/FTIR Starch.pdf]

Peak Find - starch.jws

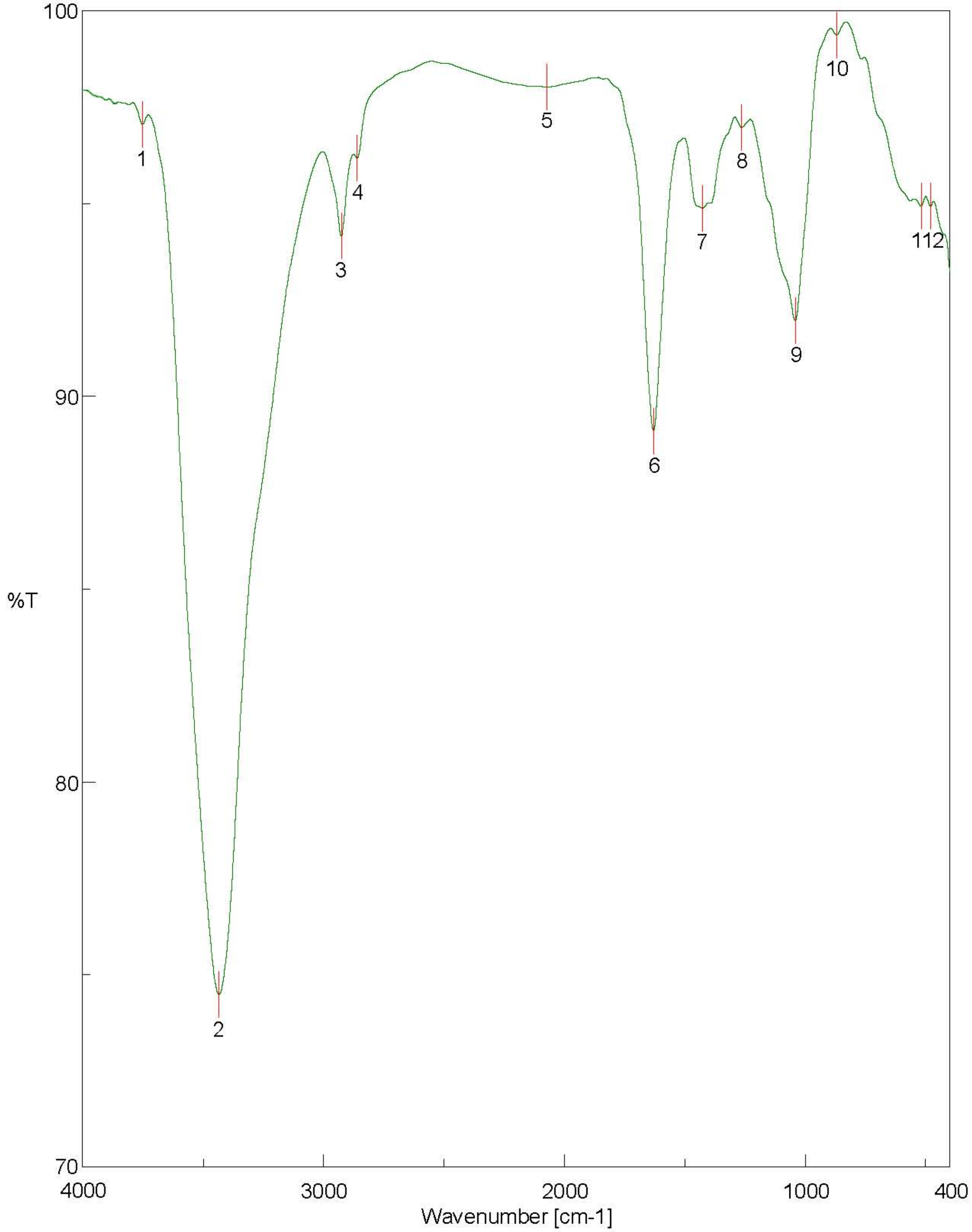

[Result of Peak Picking]

No.	Position	Intensity	No.	Position	Intensity	No.	Position	Intensity
1	3751.83	97.0559	2	3434.6	74.4789	3	2926.45	94.1539
4	2859.92	96.1732	5	2073.1	98.0152	6	1630.52	89.0989
7	1427.07	94.8756	8	1264.11	96.9734	9	1042.34	91.9544
10	870.703	99.3749	11	519.722	94.9324	12	480.188	94.9305
