# Supplementary material for: Synthesis and evaluation of protein-based biopolymer in production of silver nanoparticles as bioactive compound versus carbohydrates-based biopolymers
Source: R Soc Open Sci. 2020 Oct 21;7(10):200928. doi: 10.1098/rsos.200928 (PMC7657912; doi:10.1098/rsos.200928)
Supplement: Charts of TGA and FTIR [file rsos200928supp1.zip › TGA-IR charts/FTIR Starch-AgNPst.pdf]

Peak Find – starch-AgNPs.jws

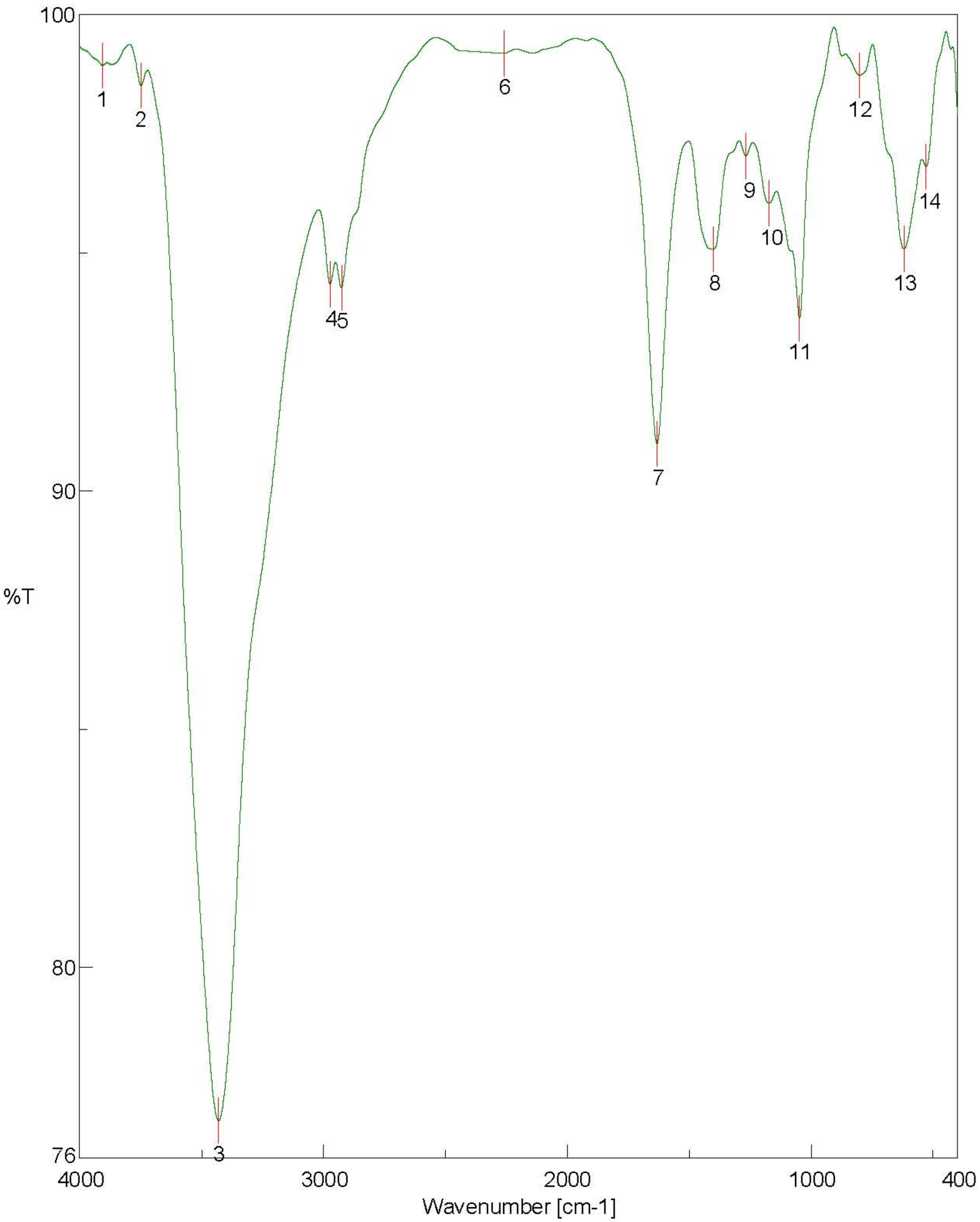

[Result of Peak Picking]

No.	Position	Intensity	No.	Position	Intensity	No.	Position	Intensity
1	3906.11	98.931	2	3747.98	98.5113	3	3430.74	76.7775
4	2972.73	94.3402	5	2926.45	94.2626	6	2261.13	99.1822
7	1632.45	90.9895	8	1401.03	95.0731	9	1267.97	97.0253
10	1173.47	96.038	11	1048.12	93.6232	12	803.206	98.7204
13	620.002	95.0824	14	529.364	96.8053			
