# Supplementary material for: Synthesis and evaluation of protein-based biopolymer in production of silver nanoparticles as bioactive compound versus carbohydrates-based biopolymers
Source: R Soc Open Sci. 2020 Oct 21;7(10):200928. doi: 10.1098/rsos.200928 (PMC7657912; doi:10.1098/rsos.200928)
Supplement: Charts of TGA and FTIR [file rsos200928supp1.zip › TGA-IR charts/TGA Carboxymethyl cellulose-AgNPs.pdf]

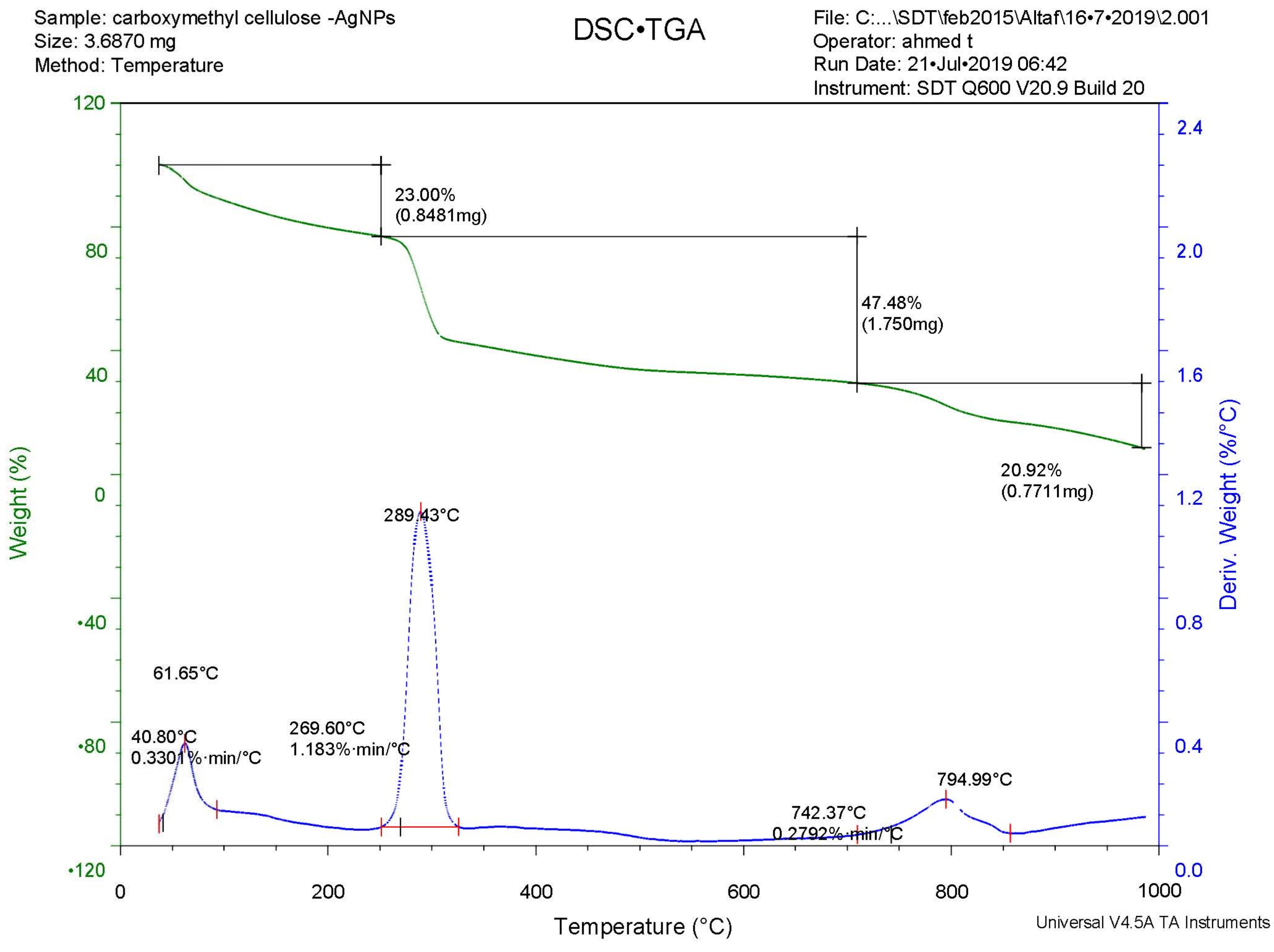
Sample: carboxymethyl cellulose -AgNPs
Size: 3.6870 mg
Method: Temperature
DSC•TGA
File: C:...\SDT\feb2015\Altaf\16•7•2019\2.001
Operator: ahmed t
Run Date: 21•Jul•2019 06:42
Instrument: SDT Q600 V20.9 Build 20
23.00%
(0.8481mg)
47.48%
(1.750mg)
20.92%
(0.7711mg)
289.43°C
61.65°C
40.80°C
0.3301%·min/°C
269.60°C
1.183%·min/°C
742.37°C
0.2792%·min/°C
794.99°C
Weight (%)
Deriv. Weight (%/°C)
Temperature (°C)
Universal V4.5A TA Instruments
