# Supplementary material for: Synthesis and evaluation of protein-based biopolymer in production of silver nanoparticles as bioactive compound versus carbohydrates-based biopolymers
Source: R Soc Open Sci. 2020 Oct 21;7(10):200928. doi: 10.1098/rsos.200928 (PMC7657912; doi:10.1098/rsos.200928)
Supplement: Charts of TGA and FTIR [file rsos200928supp1.zip › TGA-IR charts/FTIR-Oxidized cellulose.pdf]

Peak Find – oxidized cellulose.jws

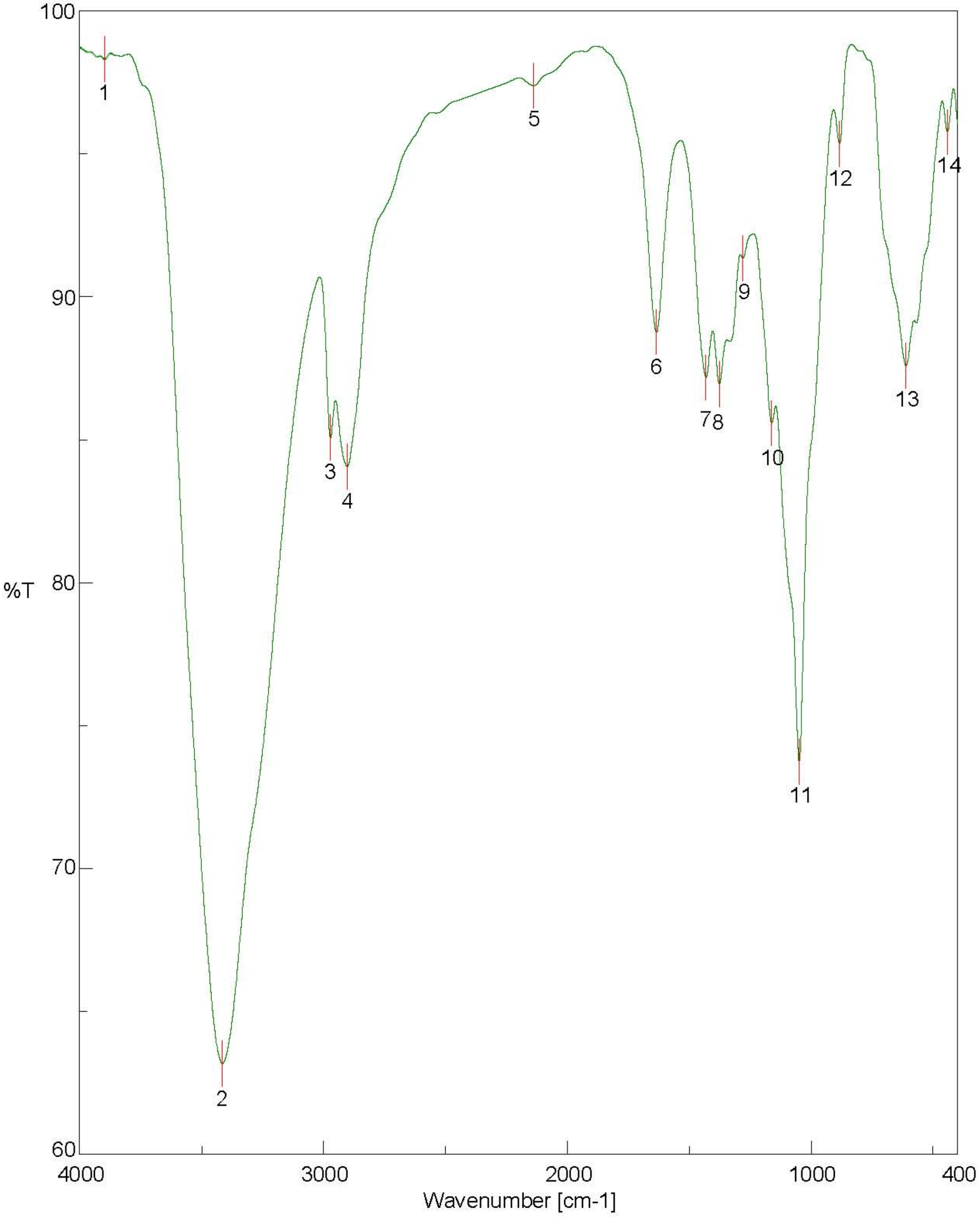

[Result of Peak Picking]

No.	Position	Intensity	No.	Position	Intensity	No.	Position	Intensity
1	3897.43	98.3159	2	3414.35	63.1564	3	2971.77	85.0689
4	2902.34	84.0464	5	2138.67	97.3828	6	1634.38	88.7614
7	1431.89	87.1659	8	1376.93	86.9384	9	1280.5	91.3457
10	1161.9	85.5712	11	1050.05	73.7286	12	884.202	95.3385
13	611.324	87.5785	14	441.619	95.7605			
