# Supplementary material for: Synthesis and evaluation of protein-based biopolymer in production of silver nanoparticles as bioactive compound versus carbohydrates-based biopolymers
Source: R Soc Open Sci. 2020 Oct 21;7(10):200928. doi: 10.1098/rsos.200928 (PMC7657912; doi:10.1098/rsos.200928)
Supplement: Charts of TGA and FTIR [file rsos200928supp1.zip › TGA-IR charts/FTIR carboxymethyl cellulose-AgNPs.pdf]

Peak Find – carboxymethyl cellulose-AgNPs.jws

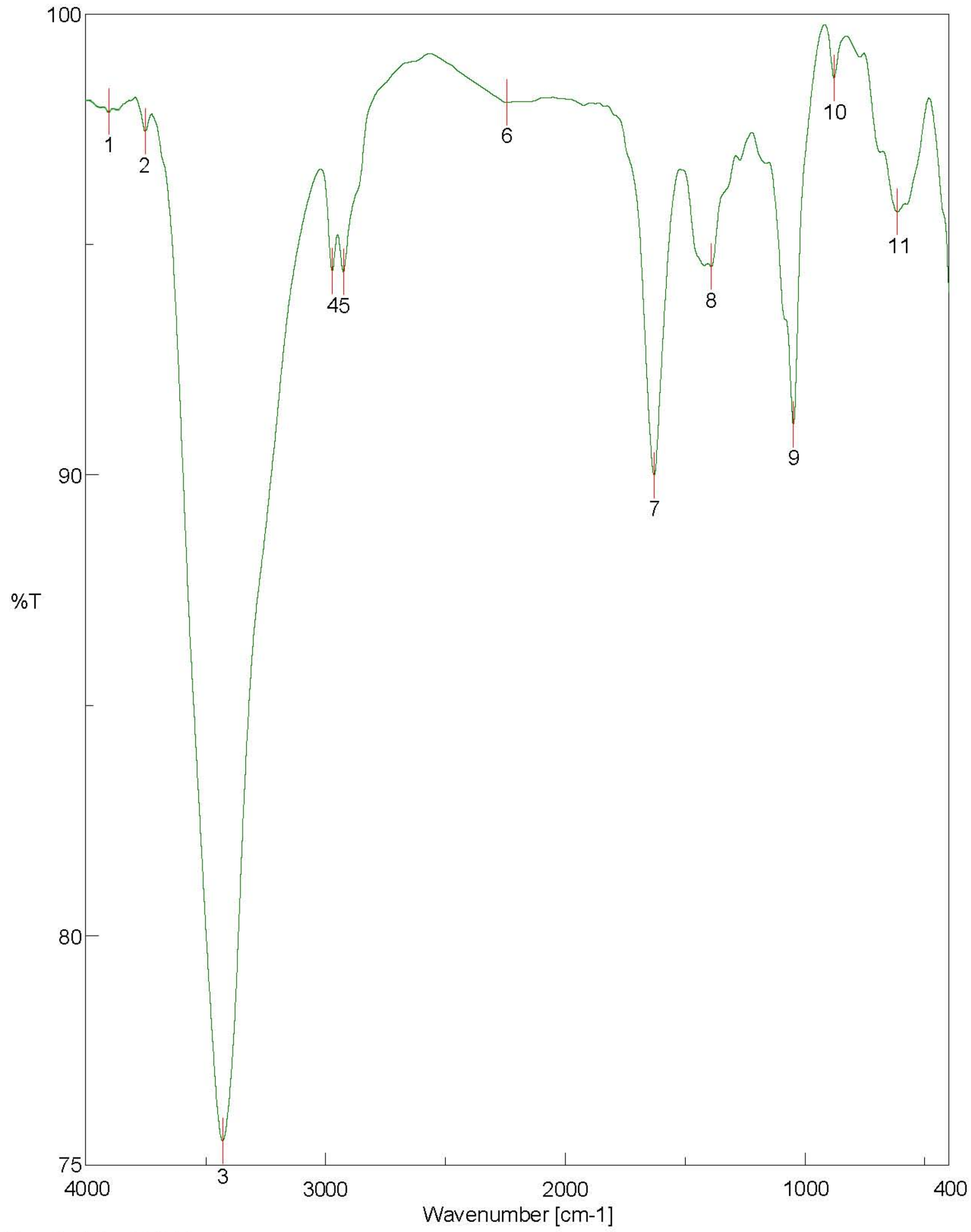

[Result of Peak Picking]

No.	Position	Intensity	No.	Position	Intensity	No.	Position	Intensity
1	3903.22	97.8833	2	3752.8	97.46	3	3428.81	75.5382
4	2972.73	94.4289	5	2925.48	94.4116	6	2245.7	98.0823
7	1629.55	89.9848	8	1391.39	94.5253	9	1049.09	91.0942
10	879.381	98.6167	11	615.181	95.7102			
