# Supplementary material for: Synthesis and evaluation of protein-based biopolymer in production of silver nanoparticles as bioactive compound versus carbohydrates-based biopolymers
Source: R Soc Open Sci. 2020 Oct 21;7(10):200928. doi: 10.1098/rsos.200928 (PMC7657912; doi:10.1098/rsos.200928)
Supplement: Charts of TGA and FTIR [file rsos200928supp1.zip › TGA-IR charts/FTIR carboxymethyl cellulose.pdf]

Peak Find –carboxymethyl cellulose.jws

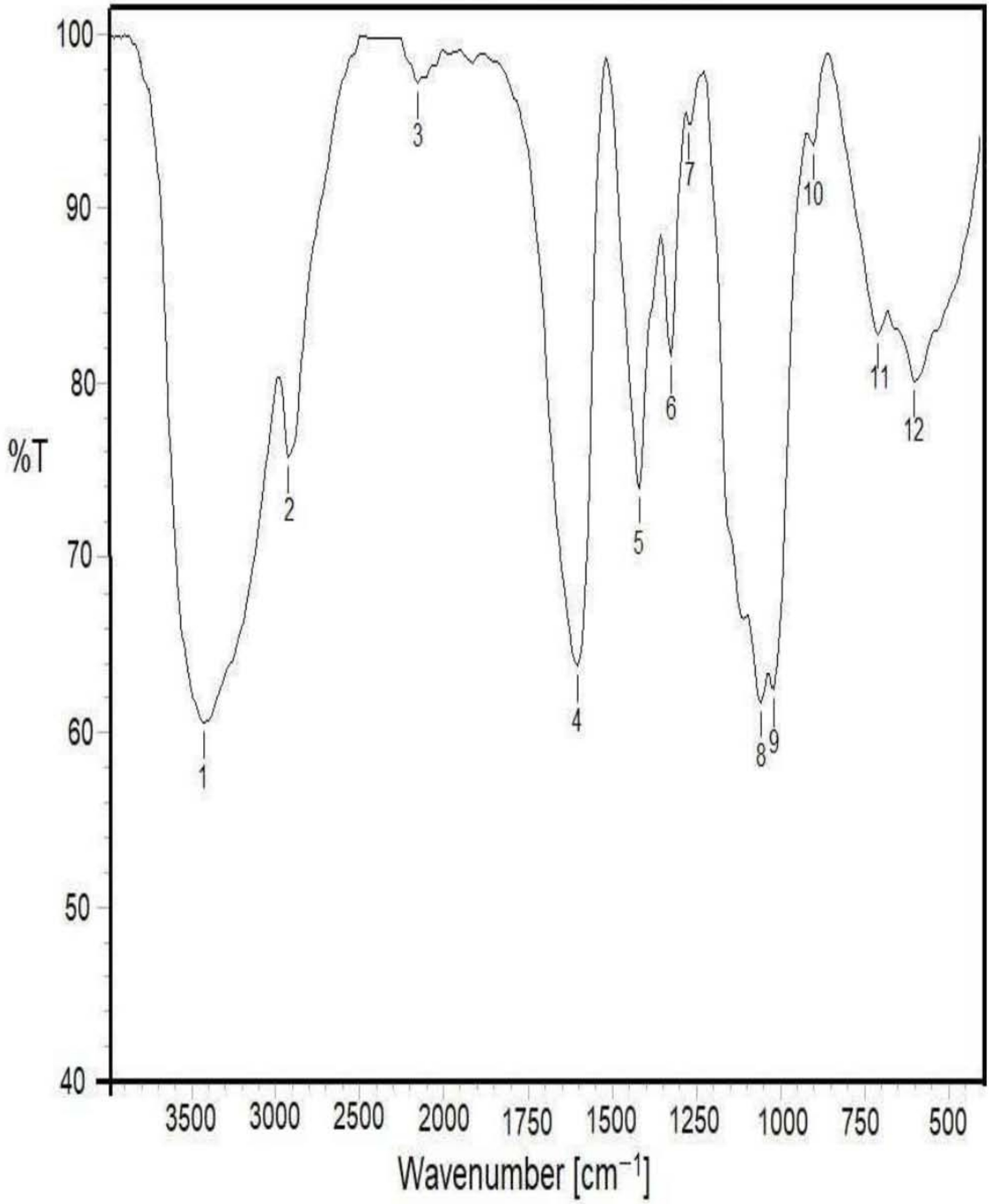

[Result of Peak Picking]

No.	Position	Intensity	No.	Position	Intensity	No.	Position	Intensity
1	3425.58	60.57896	2	2924.19	75.69708	3	2152.56	97.26354
4	1604.77	63.82687	5	1419.61	73.89718	6	1327.03	81.55754
7	1273.02	94.86636	8	1057.99	66.79749	9	1018.41	62.50372
10	902.69	93.5776	11	709.8	82.7749	12	601.79	80.08893
