# Supplementary material for: Synthesis and evaluation of protein-based biopolymer in production of silver nanoparticles as bioactive compound versus carbohydrates-based biopolymers
Source: R Soc Open Sci. 2020 Oct 21;7(10):200928. doi: 10.1098/rsos.200928 (PMC7657912; doi:10.1098/rsos.200928)
Supplement: Charts of TGA and FTIR [file rsos200928supp1.zip › TGA-IR charts/FTIR-sodium caseinate.pdf]

Peak Find – caseinate.jws

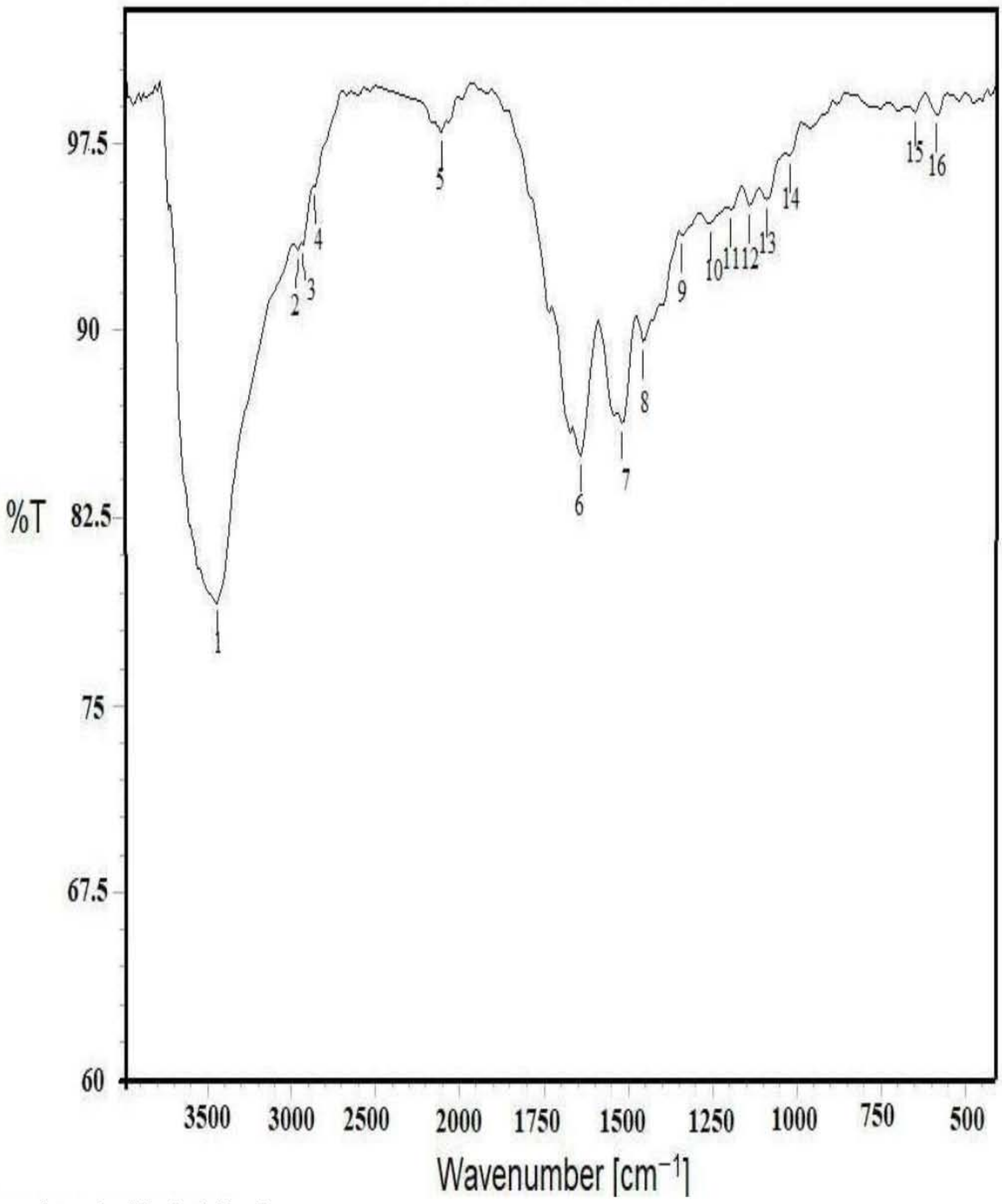

[Result of Peak Picking]

No.	Position	Intensity	No.	Position	Intensity	No.	Position	Intensity
1	3448.72	79.12447	2	2962.66	93.24594	3	2931.31	93.44035
4	2862.36	95.79242	5	2106.72	98.02214	6	1643.35	84.93196
7	1519.91	86.30546	8	1458.18	89.60589	9	1342.45	93.87736
10	1257.59	94.33131	11	1195.87	94.89228	12	1141.96	95.06386
13	1087.85	95.23719	14	1018.41	97.05737	15	648.01	98.78877
16	586.36	98.66852						
