# Supplementary material for: Synthesis and evaluation of protein-based biopolymer in production of silver nanoparticles as bioactive compound versus carbohydrates-based biopolymers
Source: R Soc Open Sci. 2020 Oct 21;7(10):200928. doi: 10.1098/rsos.200928 (PMC7657912; doi:10.1098/rsos.200928)
Supplement: Charts of TGA and FTIR [file rsos200928supp1.zip › TGA-IR charts/FTIR Oxidized cellulose-AgNPs.pdf]

# Peak Find – oxidized cellulose-AgNPs.jws

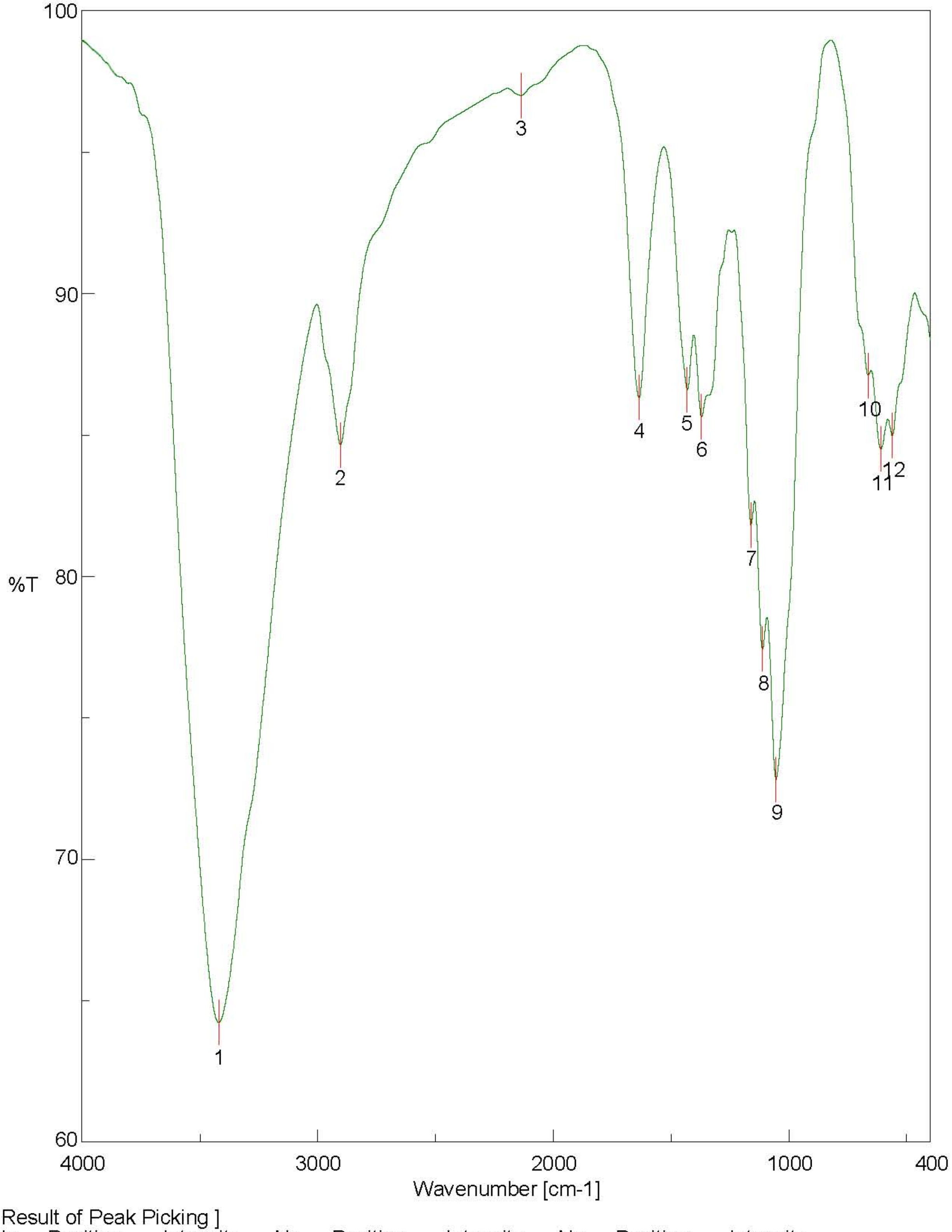


[ Result of Peak Picking ]

| No. | Position | Intensity | No. | Position | Intensity | No. | Position | Intensity |
|---|---|---|---|---|---|---|---|---|
| 1 | 3419.17 | 64.2192 | 2 | 2903.31 | 84.6498 | 3 | 2137.71 | 97.0007 |
| 4 | 1636.3 | 86.3149 | 5 | 1430.92 | 86.5904 | 6 | 1371.14 | 85.6452 |
| 7 | 1160.94 | 81.8227 | 8 | 1111.76 | 77.4242 | 9 | 1054.87 | 72.8239 |
| 10 | 663.393 | 87.1024 | 11 | 610.36 | 84.4972 | 12 | 561.184 | 84.9718 |
